# Pullulan nanofibers containing the antimicrobial palindromic peptide LfcinB (21–25)_Pal_ obtained *via* electrospinning

**DOI:** 10.1039/c9ra03643a

**Published:** 2019-07-01

**Authors:** Julieth Tatiana Román, Carlos Alberto Fuenmayor, Carlos Mario Zuluaga Dominguez, Dianney Clavijo-Grimaldo, Martha Acosta, Javier Eduardo García-Castañeda, Ricardo Fierro-Medina, Zuly Jenny Rivera-Monroy

**Affiliations:** Science Faculty, Universidad Nacional de Colombia Av. carrera 30 no. 45-03 Bogotá Zip code 111321 Colombia zjriveram@unal.edu.co +57 1 3165000 ext. 14436; Instituto de Ciencia y Tecnología de alimentos (ICTA), Universidad Nacional de Colombia Av. carrera 30 no. 45-03 Bogotá Zip code 111321 Colombia; Agricultural Sciences Faculty, Universidad Nacional de Colombia Av. carrera 30 no. 45-03 Bogotá Zip code 111321 Colombia; Medicine Faculty, Universidad Nacional de Colombia Av. carrera 30 no. 45-03 Bogotá Zip code 111321 Colombia; Servicio Nacional de Aprendizaje (SENA) Technopark Calle 54 No. 10-39 Zip code 110231 Bogotá Colombia

## Abstract

Electrospinning technology is useful for making ultrafine drug-eluting fibers for the clinical treatment of wounds. We show the incorporation of an antimicrobial LfcinB-derived peptide into Pullulan nanofibers. The palindromic peptide LfcinB (21–25)_Pal_: RWQWRWQWR was synthesized, purified, and characterized by means of the RP-HPLC and MALDI-TOF MS methods. The peptide's antibacterial activity against the *E. coli* ATCC 25922 strain was evaluated, and the peptide LfcinB (20–25)_Pal_ exhibited significant antibacterial activity. Nanofibers were obtained by electrospinning a Pullulan or Pullulan-LfcinB (21–25)_Pal_ solution. The obtained nanofibers were characterized *via* microscopy (AFM and SEM) and RP-HPLC chromatography. The peptide incorporation efficiency was 31%. The Pullulan-LfcinB (21–25)_Pal_ nanofibers were soluble in water, and the peptide was liberated immediately. The Pullulan-LfcinB (21–25)_Pal_ nanofibers exhibited the same antibacterial activity against *E. coli* strain as the free peptide LfcinB (21–25)_Pal_. The results suggest that Pullulan-LfcinB (21–25)_Pal_ nanofibers could be considered for designing and developing antibacterial wound dressings.

## Abstract 2

Pullulan nanofibers containing antimicrobial peptide derived from LfcinB an option as biomaterial for wound dressings.

## Graphical abstract

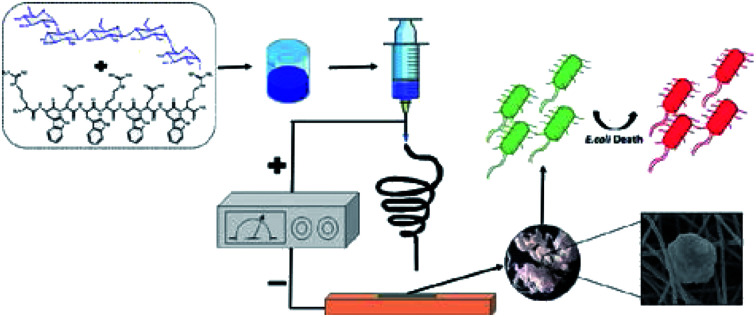

## 1 Introduction

Bacterial infections are frequent in wounds, causing complications in patients that increase morbidity, mortality, and treatment costs.^1–3^ Approximately 50–60% of deaths caused by paediatric skin burns are related to wound infections.^1–3^ Despite advances in techniques, surgical materials, and sterilization methods, infectious wounds continue to be a major problem in the course of treatments.^4^ Wound treatment involves procedures such as cleaning and disinfection. Topical or systemic prophylactic antimicrobial treatment is complemented with bandages and/or skin grafts.^5,6^ The wound dressing must be innocuous and must protect the wound while allowing a humid environment, accelerating the wound closure and preventing infections.^6^ Most of the wound dressings available in the market are made of cotton, cloth, and gauze, and some more specialized ones consist of silicone meshes.^7,8^ However, these dressings have limited air permeability and a low swelling, and are difficult to use in the topical administration of drugs.^9^ Furthermore, dressing replacement can cause skin removal, resulting in skin pain and wound irritation. Therefore, the design and development of new therapeutic dressings for wound treatment is of great significance.^9^ Nanofibers have attracted recent interest in drug administration since they have a structure similar to the human extracellular matrix and could be a protection barrier that allows gas exchange. Nanofibers have a huge potential for food, biomedical, and engineering applications, because of their high surface area, excellent mechanical properties, and capacity and versatility for surface modification.^10,11^ Over the last few decades, nanomaterials from natural or/and synthetic polymers have been used for developing drug administration systems.

Pullulan is a polymer produced from the industrial fermentation of starch syrup caused by *Aureobasidium pullulans*.^12,13^ This polymer possesses a linear structure consisting of three glucose units connected by -1,4 glycosidic bonded maltotriose that are linked *via* -1,6 glycosidic linkage with a molecular weight close to 2000 kDa. Pullulan is a biocompatible and biodegradable material that is used in pharmaceuticals, cosmetics, and the food industry.^12,13^ Pullulan is a white powder, odorless, tasteless, highly water soluble, and stable under temperature and pH changes. The main advantages for its use as a vehicle for transporting drugs are that it is a blood-compatible polymer and is not toxic, immunogenic, mutagenic, or carcinogenic.^12,13^ Electrospinning is a low-cost, operationally simple, and fast technique that can be performed at room temperature.^14,15^ It is a simple and versatile method for obtaining dynamic and highly porous nanofibers with a high surface area and good mechanical properties.^15,16^ This technique consists of the application of a strong electric field to the polymer solution held by its surface tension to the end of a capillary tube. Charge is induced on the liquid surface, and mutual charge repulsion causes a force directly opposite to the surface tension.^14–18^ As the intensity of the electric field is increased, the hemispherical surface of the solution at the tip of the capillary tube elongates to form a conical shape known as the Taylor cone.^14–18^ When the electric field reaches a critical value at which the repulsive electric force overcomes the surface tension force, a charged jet of the solution is ejected from the tip of the Taylor cone. Because this jet is charged, its trajectory can be controlled by an electric field. As the jet travels in air, the solvent evaporates, leaving behind a charged polymer fiber that lies randomly on a collecting metal screen.^14–18^ The electrospinning technique has been used for producing nanofibers combined with polymers, peptides, antibiotics, anticancerigenic drugs, proteins, ADN, viable cells, essential oils, *etc.*^9,14,19–21^

Antimicrobial peptides (AMPs) are molecules that belong to the innate immune response of organisms. AMPs exhibit activity against Gram-positive and Gram-negative bacteria, fungi, viruses, and parasites.^22,23^ AMPs are effective, stable, safe molecules, and they are less prone to induce resistance, so they are considered to be a viable alternative for developing new therapeutic agents.^22,23^ Bovine lactoferricin (LfcinB) is an AMP with antibacterial, antifungal, antiparasitic, and anticancerigenic activity.^24,25^ It has been proposed that LfcinB interacts electrostatically with the bacterial membrane, *i.e.* between positive charges of LfcinB side chains with the negative charges on the bacterial surface. So the hydrophobic side chains of the peptide interact with the membrane lipid, causing its disruption and leading to cellular lysis.^24,25^ The RRWQWR sequence is the minimal motif of LfcinB with antimicrobial activity.^26^ In previous reports, it has been demonstrated that short synthetic peptides exhibited higher antibacterial, antifungal, and anticancerigenic activity than LfcinB.^26–31^ The palindromic peptide LfcinB (21–25)_Pal_: RWQWRWQWR is a promising peptide for developing new therapeutic agents.^26–30^ This peptide exhibits antibacterial activity against Gram-positive and Gram-negative bacteria and fungi and a cytotoxic effect against oral carcinoma and breast cancer cell lines.^26–31^ The results obtained in this investigation suggest that it is possible to obtain Pullulan nanofibers containing the peptide LfcinB (21–25)_Pal_ (Pullulan-LfcinB (21–25)_Pal_) by means of electrospinning. The Pullulan-LfcinB (21–25)_Pal_ nanofibers were characterized, and their antibacterial activity against *E. coli* strain was the same as that of the peptide LfcinB (21–25)_Pal_. This work demonstrated that it is possible incorporate antimicrobial peptides to the Pullulan nanofibers, and the Pullulan/peptide nanofibers liberates the peptide in instantaneous manner. Our results suggest that nanofibers based on Pullulan/peptide could be considered for designing wound dressing as an administration system in the skin burn treatment.

## 2 Experimental details

### 2.1 Reagents

Mueller–Hinton, agar SPC, Mueller–Hinton Broth (MHB), ciprofloxacin (Bayer), and bacterial strains were obtained from ATCC (Manassas, VA, USA). Rink Amide resin, Fmoc-amino acids, dicyclohexylcarbodiimide (DCC), and 1-hydroxy-6-chlorobenzotriazol (6-Cl-HOBT) were purchased from AAPPTEC (Louisville, KY, USA). Methanol, diethyl ether, acetonitrile (ACN), trifluoroacetic acid (TFA), dichloromethane (DCM), diisopropylethylamine, *N*,*N*-dimethylformamide (DMF), ethanedithiol (EDT), isopropanol (IPA), methanol, and triisopropylsilane (TIS) were purchased from Merck (Darmstadt, Germany). Silicycle® SiliaPrep™ C18 cartridges were kindly donated by EcoChem Especialidades Químicas. Food-grade Pullulan (PF-20 Grade, 200 kDa) was purchased from Hayashibara Biochemical Laboratories Inc. (Okayama, Japón) and donated by Dr Giusto Faravelli (Milan, Italy). All the reagents were used without further purification.

### 2.2 Peptide synthesis

The peptide was synthesized using solid-phase peptide synthesis (SPPS) and the Fmoc/*t*Bu strategy (SPPS-Fmoc/*t*Bu).^32^ Briefly, Rink Amide resin (0.046 meq g^−1^) was swollen in DMF for 24 h at room temperature (RT). Fmoc group removal was carried out by the treatment of resin or resin-peptide with 2.5% 4-methylpiperidine in DMF for 10 min at RT. Amino acid coupling reactions were carried out twice, using a 5 molar excess with respect to the resin milliequivalents. First, the Fmoc-amino acid was mixed with DCC/6-Cl-HOBt (1 : 1 : 1) in 2 mL of DMF, and the mixture was gently shaken for 15 min at RT. Then preactivated Fmoc amino acid was added to the resin or resin-peptide, and the reaction mixture was stirred at RT for 3 h. When the coupling reaction was not complete the procedure was repeated until the Kaiser test was negative. Fmoc-group removal and coupling reactions were monitored using the Kaiser test. Side-chain deprotection reactions and peptide separation from the solid support were carried out *via* the treatment of the resin-peptide with a solution containing TFA/water/TIS/EDT (93/2/2.5/2.5 v/v) for 4 h at RT. Then the reaction was filtrated, the crude peptide was precipitated with ethylic ether, and the solid was washed five times with ether and dried at RT.

### 2.3 Reverse-phase HPLC

RP-HPLC analysis was performed on a Chromolith® C-18 (50 × 4.6 mm) column using an Agilent 1200 liquid chromatograph (Omaha, Nebraska, USA) with UV-Vis detector (210 nm). For the analysis of the peptides (10 μL, 1 mg mL^−1^), a linear gradient was applied from 5% to 50% solvent B (0.05% TFA in acetonitrile) in solvent A (0.05% TFA in water) with a gradient time of 8 min. The flow rate was 2.0 mL min^−1^ at room temperature.

### 2.4 Peptide purification

The peptides were purified using RP-SPE columns^33,34^ (Silicycle® Siliaprep™ C18, 5 g, 40–60 μm). The columns were activated and equilibrated in accordance with manufacturer recommendations. The peptides were dissolved in A (100 mg mL^−1^), loaded into the column and eluted with a solvent B gradient. The fractions were analyzed *via* RP-HPLC chromatography, and those that contained the pure peptide were collected and lyophilized.

### 2.5 MALDI-TOF MS

The peptide (1 mg mL^−1^) was mixed with the matrix (1.0 mg mL^−1^ of 2,5-dihydroxybenzoic acid, or sinapinic acid) (2 : 18, v/v), and then 1 μL of this mixture was seeded on a steel target. The experiment was carried out on a Ultraflex III TOF-TOF mass spectrometer (Bruker Daltonics, Bremen, Germany), Laser: 250 shots and 25–30% power.

### 2.6 Electrospinning assays

The nanofibers were obtained by electrospinning of a Pullulan or Pullulan-peptide solution. The optimal ratio between Pullulan and peptide was established as the maximum peptide quantity that didn't affect the Pullulan spinability. It was found that Pullulan/peptide 74 : 1 w/w was the best ratio and being 20% w/w the final concentration Pullulan. (a) Pullulan (20% w/v) was dissolved in deionized water. (b) Pullulan (975 mg mL^−1^) and LfcinB (20–25)_Pal_ peptide (13.2 mg mL^−1^) solutions were mixed and diluted with deionized water until reaching a Pullulan concentration of 20% w/v. The solution was stored overnight at 4 °C. After that, the solution was passed through a syringe (KDS100; KD-Scientific, New Hope, PA). The caudal was 0.5 mL h^−1^, the applied voltage (Spellman SL150) was 15 kV, and the distance from the needle to the collector was 11 cm. Once the Taylor cone was formed, the nanofibers were collected on aluminium foil for 20 min.^14^ The membranes were sterilized with UV radiation and stored in a desiccant chamber.

### 2.7 Kaiser test

The presence of free amine groups in the nanofibers was evaluated using the Kaiser test.^35^ Briefly, (1) phenol was dissolved in absolute ethanol (4 g mL^−1^); (2) 1.0 mL of KCN aqueous solution (0.65 mg mL^−1^) was mixed with 49.0 mL of pyridine; (3) 1.25 g of ninhydrin was dissolved in 25 mL of absolute ethanol; (4) solutions (1) and (2) were mixed (1 : 1 v/v), solutions 3 and 4 were mixed (1 : 2; v/v), and then (i) Pullulan-LfcinB (21–25)_Pal_ nanofiber (1 mg) or (ii) Pullulan nanofiber (1 mg) was added. The reaction mixture was heated at 105 °C for 5 min. A blue solution indicates a positive test for the presence of free amine groups, while a yellow solution indicates a negative test.

### 2.8 Encapsulation efficiency

The quantity of peptide in the nanofibers was determined *via* RP-HPLC chromatography. Briefly, Pullulan nanofibers (0.18% w/w) or Pullulan-LfcinB (21–25)_Pal_ nanofibers (2.36% w/w) were dissolved in solvent A and passed through a Merck Chromolith® C18 (50 × 4.6 mm) column, as described in 2.3.

### 2.9 Atomic force microscopy (AFM)

The nanofibers were put on the metallic support (1 cm diameter) of the microscope (nano surf easyScan 2 flexAFM). The images were obtained while varying the size of the analysis region (100, 50, 10, and 5 μm), and the force was 20–25 N, 256 points and 799 ms by line.^36^

### 2.10 Scanning electronic microscopy (SEM)

The nanofibers were analyzed *via* SEM microscopy (TESCA, VEGA 3, and secondary electron detection). Gold-covered nanofibers were put on the metallic support (1 cm diameter) of the microscope, and the images were obtained while varying the size of the analysis region (50, 10, 5, 2, and 1 μm). The support movement was random. The distribution of the diameter of the fibers was determined by measuring 100 fibers in the whole membrane.

### 2.11 Antibacterial activity assays

The minimum inhibitory concentration (MIC) was determined using a microdilution assay.^26,29^ In brief, using a 96-well microtiter plate, 90 μL of peptide (200, 100, 50, 25, 12.5, and 6.2 μg mL^−1^) or 90 μL of Pullulan-LfcinB (21–25)_Pal_ (33 mg/250 μL) were diluted serially until the final peptide concentration in each well was 200, 100, 50, 25, 12.5, and 6.2 μg mL^−1^, and 10 μL of inoculum (5 × 10^6^ CFU mL^−1^) was added to each well. Afterward, the bacteria were incubated for 24 h at 37 °C and the absorbance at 620 nm was measured. The minimum bactericidal concentration (MBC) was determined as follows: a small sample was taken from each well where there was no visible growth, using an inoculation loop, which was then spread on MHA plates and incubated overnight at 37 °C. MBC was considered to be the plate that exhibited no bacterial growth. Each of these tests was performed twice (*n* = 2).

### 2.12 Statistical analysis

All the results were analyzed *via* SEM, expressed as the standard error of the mean, and the statistical analysis was carried out *via* one-way analysis of variance (ANOVA). A *p*-value less than 0.05 was considered statistically significant.

## 3 Results and discussion

### 3.1 Peptide synthesis

**Fig. 1:**
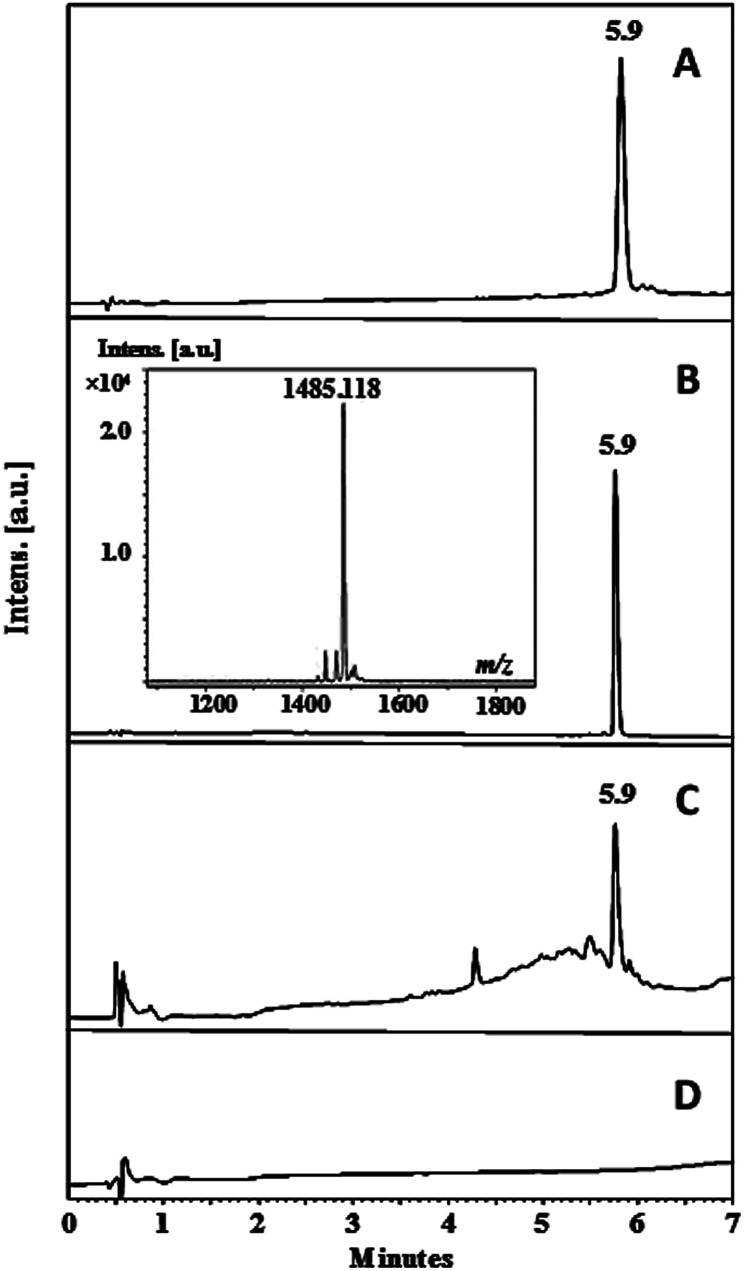
Chromatographic characterization of the peptide and membranes. Peptide LfcinB (21–25)_Pal_ (A) crude, (B) pure; (C) Pullulan-LfcinB (21–25)_Pal_ membrane and (D) Pullulan membrane (control).

Pullulan nanofibers containing the palindromic peptide LfcinB (21–25)_Pal_ (Pullulan-LfcinB (21–25)_Pal_) were obtained by means of the electrospinning technique. For this, peptide LfcinB (21–25)_Pal_ was synthesized using the SPPS and Fmoc/*t*Bu methods. The LfcinB (21–25)_Pal_ peptide synthesis was fast and the incorporation of amino acids to the growing chain was efficient. In the synthesis of RWQWRWQWR peptide, only Arg and Gln amino acids (in bold and underline) required two coupling reactions to attach the amino acid completely. The efficiency of coupling reaction depends mainly of the steric hindrance which difficult the access of pre-activated amino acid to amine groups of growing chain. While the Fmoc removal reaction, side chain deprotection, and peptide separation from the solid support proceeded in a satisfactory manner. The crude peptide chromatographic profile exhibited a main signal (*t*_R_: 5.9 min; purity 86%) suggesting that the peptide synthesis was efficient, making the purification process easy and fast (Fig. 1A). The crude peptide was purified *via* RP-SPE chromatography and characterized *via* RP-HPLC and MALDI-TOF MS.^33^ The chromatographic profile of the pure peptide exhibited a main signal with the same *t*_R_ as the crude peptide product, and the chromatographic purity was 97% (Fig. 1B). The peptide purity increased from 86% to 97%, indicating that the purification process was efficient. The pure peptide had the expected *m*/*z* corresponding to [M + H^+^] specie (Table 1 and Fig. 1B). The chromatographic characterization of Pullulan membrane (was used as control) and Pullulan-LfcinB (21–25)_Pal_ was carried out using RP-HPLC. Pullulan-LfcinB (21–25)_Pal_ membrane's chromatographic profile exhibited a main signal with the same *t*_R_ as the peptide LfcinB (21–25)_Pal_ (Fig. 1B and C), while the Pullulan membrane's (control) chromatographic profile did not exhibit any signal. However, the Pullulan-LfcinB (21–25)_Pal_ membrane's chromatographic profile exhibited minor signals at *t*_R_ less than 5.9 min, suggesting that the peptide elution was affected by the presence of the Pullulan membrane.

Peptide and Pullulan-LfcinB (21–25)_Pal_ nanofibers characterization and antimicrobial activity. Electrospinning conditions

**Table:** 

Peptide	MS *m*/*z* [M + H]^+^		RP-HPLC analysis		Antimicrobial activity CMI/CMB **μM** (μg mL^−1^)
	Theoretical	Experimental	*t*_R_ (min)	aPurity	*E. coli* ATCC 25922
LfcinB (21–25)_Pal_	1485.75	1485.12	5.9	97	**17**/**34**(50)
Pullulan-LfcinB (21–25)_Pal_	—	—	5.9	NA	**17**(25)/**68**(100)

a Peptide purity was calculated using the percentage of peak area at the chromatographic profile.

**Table:** 

	Electrospinning conditions			LfcinB (21–25)_Pal_ concentration (ppm)		Recovery (%)
	*V* (kV)	Flow rate (mL h^−1^)	Recollection time (min)	Solution electrospinning	Nanofiber	
Pullulan-LfcinB (21–25)_Pal_	15	0.5	20	13 347	1600	12
					4100	31

### 3.2 Membrane characterization

**Fig. 2:**
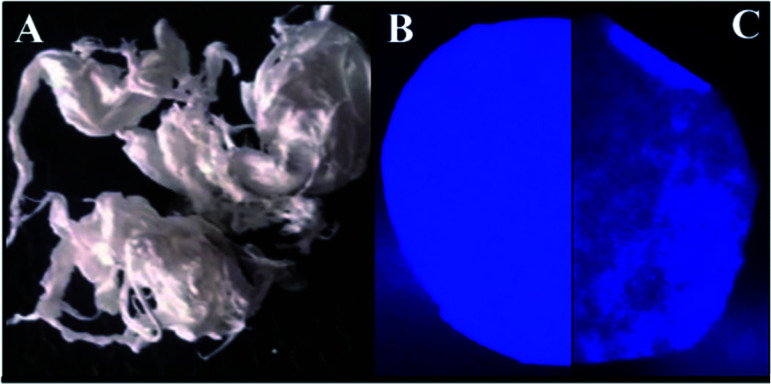
Membrane characterization. (A) Pullulan-LfcinB (21–25)_Pal_ membrane photography. Membranes were observed under UV radiation at 254 nm: (B) Pullulan-LfcinB (21–25)_Pal_, and (C) Pullulan membranes.

The Pullulan-LfcinB (21–25)_Pal_ membrane Kaiser test was positive (blue stain), indicating that this membrane contains primary amine groups from the side chains (Arg) and *N*-terminal of the peptide, while the Pullulan membrane Kaiser test was negative (yellow stain), which is in accordance with what was expected. When the membranes were observed under UV radiation (254 nm), only the Pullulan-LfcinB (21–25)_Pal_ membrane exhibited fluorescence, possibly due to chromophore groups from the peptide side chains of Trp, Arg and the peptidyl bond (Fig. 2B).

Pullulan and Pullulan-LfcinB (21–25)_Pal_ membranes exhibited a similar aspect, smooth texture, white color, good mechanical stability, homogeneous surface, and easy manipulation (Fig. 2). Pullulan-LfcinB (21–25)_Pal_ membranes exhibited diameters between 7.35 and 8.04 cm and an average weight of 12 mg. Pullulan membrane diameters were between 7.57 cm and 7.36 cm with an average weight of 13.2 mg. In both cases, the membranes were highly soluble in water, in accordance with the high solubility of both the Pullulan and the peptide in water. The Pullulan-LfcinB (21–25)_Pal_ membrane's properties are in accordance with those required for wound dressings. These membranes may act as a vehicle for peptide application over the wound, membrane removal being unnecessary. This implies less contact with the wound due to easy manipulation, which increases the patient's wellness.

**Fig. 3:**
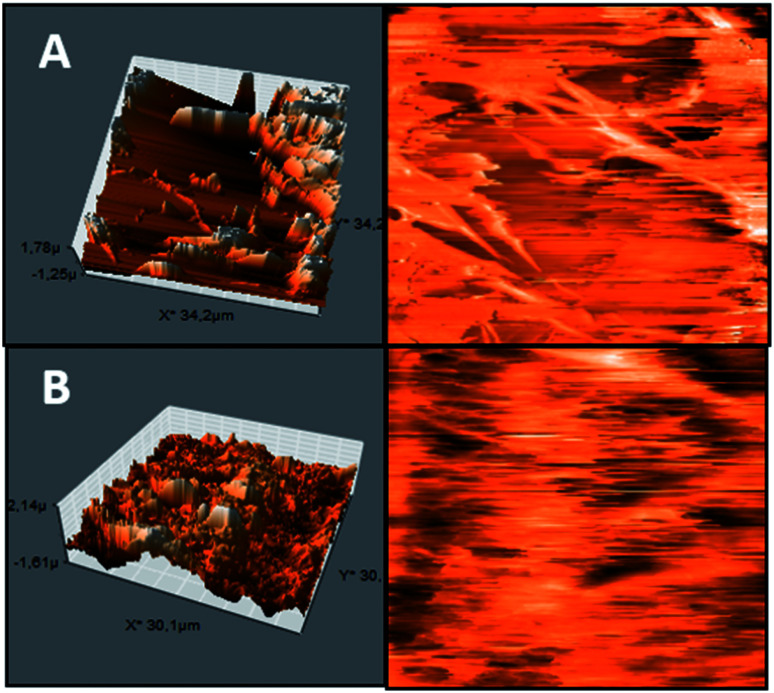
AFM membrane. (A) Pullulan-LfcinB (21–25)_Pal_ and (B) Pullulan membranes. (Right) membrane 3D projection; (left) membrane topographic profile.

The Pullulan-LfcinB (21–25)_Pal_ membrane's surface exhibited changes in surface morphology compared with the Pullulan membrane's surface. Some zones exhibited peak-to-valley roughness with high peaks and great depth. The Pullulan membrane's surface exhibited more homogeneity and differences with respect to the Pullulan-LfcinB (21–25)_Pal_ membrane's surface (Fig. 3).

### 3.3 Nanofiber characterization

**Fig. 4:**
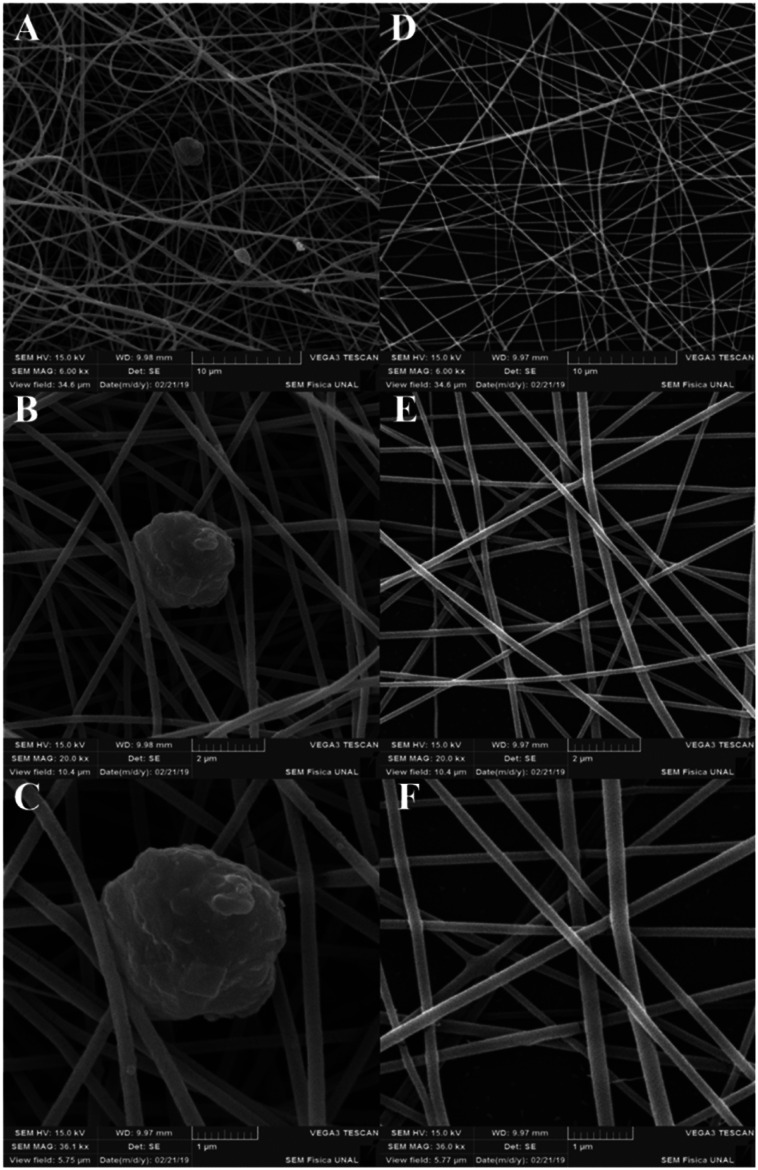
Nanofiber characterization *via* SEM. (A)–(C) Pullulan-LfcinB (21–25)_Pal_ nanofibers. (D)–(F) Pullulan nanofibers.

The obtained nanofibers' morphology and structure were analyzed *via* SEM. The Pullulan and Pullulan-LfcinB (21–25)_Pal_ nanofibers' surface is smooth and uniform, suggesting that the incorporation of the peptide does not induce drastic changes in the Pullulan nanofibers (Fig. 4). The Pullulan nanofibers did not exhibit beads, which is in accordance with previous studies that showed that when the Pullulan concentration was higher than 15% w/w, the nanofibers do not exhibit bead formation after the electrospinning process.^37^

**Fig. 5:**
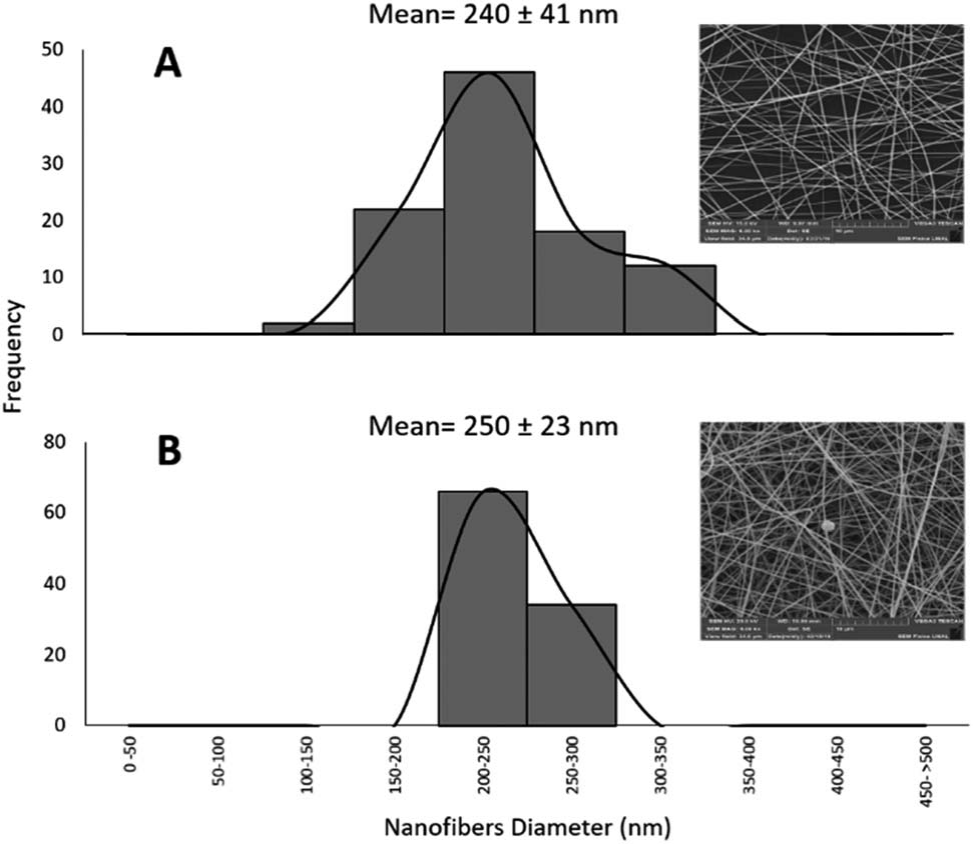
Frequency histogram of nanofiber diameter distribution. (A) Pullulan nanofibers; (B) Pullulan-LfcinB (21–25)_Pal_ nanofibers. The ANOVA indicates that there is a significant difference between the diameter of the Pullulan nanofibers and that of the Pullulan-LfcinB (21–25)_Pal_ nanofibers, (*n* = 100).

Pullulan-LfcinB (21–25)_Pal_ nanofibers exhibited some beads (nanofiber deformations), possibly formed by the peptide presence in the solution during the electrospinning process (Fig. 5). The presence of beads in Pullulan-LfcinB (21–25)_Pal_ nanofibers could be due to changes in Pullulan spinability caused by factors such as the Pullulan concentration and the presence of polymers and/or charged species in the electrospinning solution. Pullulan/PVA fibers exhibited beads between the fibers, which were caused by an increase in the routine concentration.^38^ On the other hand, Pullulan/pectine nanofibers exhibited beads when the Pullulan concentration was higher than 15%.^38^ It has been suggested that the presence of charged molecules in the solution influences bead formation in nanofibers due to the movement of static charges into the jet during the electrospinning process.^37^

Also, electrospinning products from peptides containing the motif LKLK exhibited nano-beads and nano-beads-on a string. Nano-beads on a string structure is considered to be an intermediate stage in fiber formation. This could be attributed to the development of jet instability and the capillary breakup of the electrospun peptide jet.^39^ Previous reports suggest that positively-charged side chains of peptide possibly modify the force and spinability of Pullulan.^40^ Furthermore, spinability is associated with such solution properties as molecular entanglement, surface tension, and electric conductivity, which may be affected by the presence of the peptide.

Pullulan-LfcinB (21–25)_Pal_ and Pullulan nanofibers exhibited similar mean fiber diameters; however, the fiber diameter distribution for Pullulan-LfcinB (21–25)_Pal_ (200–300 nm) was less than for Pullulan nanofibers (150–300 nm) (Fig. 5). The obtained nanofiber diameters were similar to those reported by other authors; for example, the nanofibers obtained from Pullulan 15% w/w solution exhibited diameters of 217 nm.^41^ On the other hand, the diameter values obtained by us are less than those observed for Pullulan/PVA nanofibers (312–334 nm; 400–800 nm) and Pullulan containing limonene (370 nm),^14,16,40^ while Pullulan/PEC (135 nm) or Pullulan/alginate nanofibers exhibited dimeters between 57 and 110 nm, suggesting that the incorporation of substances into the Pullulan solution decreases the fiber diameter.^38,42^ It has been established that the nanofiber diameter may be affected by electrospinning parameters such as the Pullulan concentration, the presence of charged molecules, the applied voltage, the spinning flow rate, and the receiving distance.^43^

### 3.4 Antimicrobial activity

Infections in surgical and burning wounds are caused mainly by *E. coli* and *S. aureus* strains. Antibacterial activity against the *E. coli* strain of peptide LfcinB (21–25)_Pal_, Pullulan-LfcinB (21–25)_Pal_, and Pullulan nanofibers was evaluated. The antibacterial activity assays showed that the peptide LfcinB (21–25)_Pal_ exhibited high antibacterial activity against *E. coli* ATCC 25922 (17 μM) (Table 1).

**Fig. 6:**
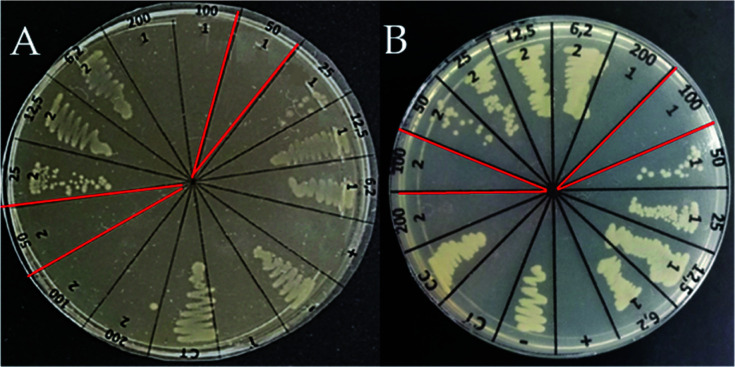
Membrane antibacterial activity. Determination of MBC of Pullulan-LfcinB (21–25)_Pal_ nanofibers and the peptide LfcinB (21–25)_Pal_ (control). Bacterial growth inhibition of the *E. coli* strain treated with (A) peptide LfcinB (21–25)_Pal_ and (B) Pullulan-LfcinB (21–25)_Pal_ nanofibers. (*n* = 2).

This MIC value was similar to other MIC values reported previously. Furthermore, this peptide exhibited antibacterial activity against other bacteria, such as *E. faecalis* ATCC 29212 (MIC = 27 μM), *S. entiriditis* ATCC 13076 (MIC = 17 μM), *S. aureus* (MIC = 135 μM), and *P. aeruginosa* ATCC (MIC = 67 μM).^26,29^ These reports suggest that the peptide LfcinB (21–25)_Pal_ could be promising for preventing wound infections caused by Gram-positive and Gram-negative bacteria. The Pullulan-LfcinB (21–25)_Pal_ nanofibers' antibacterial activity was evaluated. The MIC value of Pullulan-LfcinB (21–25)_Pal_ nanofibers was similar to the MIC value of the free peptide LfcinB (21–25)_Pal_. Fig. 6 shows that both the peptide LfcinB (21–25)_Pal_ and Pullulan-LfcinB (21–25)_Pal_ nanofibers completely inhibited the bacterial growth at 50 μg mL^−1^ and 100 μg mL^−1^, respectively.

Our results indicate that the electrospinning process of the peptide LfcinB (21–25)_Pal_/Pullulan solution does not affect the peptide's antibacterial activity.

## 4 Conclusions

We established electrospinning parameters to obtain Pullulan nanofiber containing the peptide LfcinB (21–25)_Pal_. The Pullulan-LfcinB (21–25)_Pal_ and Pullulan nanofibers exhibited a similar appearance and similar mechanical properties. The peptide incorporated into Pullulan-LfcinB (21–25)_Pal_ nanofibers and the LfcinB (21–25)_Pal_ peptide showed the same antibacterial activity against the *E. coli* strain. The electrospinning process does not affect the peptide's integrity, and the peptide is liberated instantaneously from the nanofibers. This paper constitutes a contribution to the design of wound dressing as an administration system in the treatment of skin wounds.

## Conflicts of interest

There are no conflicts to declare.

## Supplementary Material
